# Aquaporin 1 suppresses apoptosis and affects prognosis in esophageal squamous cell carcinoma

**DOI:** 10.18632/oncotarget.25722

**Published:** 2018-07-06

**Authors:** Yuzo Yamazato, Atsushi Shiozaki, Daisuke Ichikawa, Toshiyuki Kosuga, Katsutoshi Shoda, Tomohiro Arita, Hirotaka Konishi, Shuhei Komatsu, Takeshi Kubota, Hitoshi Fujiwara, Kazuma Okamoto, Mitsuo Kishimoto, Eiichi Konishi, Yoshinori Marunaka, Eigo Otsuji

**Affiliations:** ^1^ Division of Digestive Surgery, Department of Surgery, Kyoto Prefectural University of Medicine, Kyoto, 602-8566, Japan; ^2^ Department of Gastrointestinal, Breast & Endocrine Surgery, Faculty of Medicine, University of Yamanashi, Chuo, 409-3898, Japan; ^3^ Department of Pathology, Kyoto Prefectural University of Medicine, Kyoto, 602-8566, Japan; ^4^ Departments of Molecular Cell Physiology and Bio-Ionomics, Graduate School of Medical Science, Kyoto Prefectural University of Medicine, Kyoto, 602-8566, Japan; ^5^ Japan Institute for Food Education and Health, St. Agnes’ University, Kyoto, 602-8013, Japan

**Keywords:** AQP1(aquaporin 1), esophageal squamous cell carcinoma, apoptosis, death receptor signaling, cellular physiology

## Abstract

Aquaporin 1 (AQP1) is a membrane protein whose main function is to transfer water across cellular membranes. Recent studies have described important roles for AQP1 in epithelial carcinogenesis and tumor behavior. The objectives of the present study were to investigate the role of AQP1 in the regulation of genes involved in tumor progression and the clinicopathological significance of its expression in esophageal squamous cell carcinoma (ESCC). An immunohistochemical analysis was performed on 50 primary tumor samples underwent esophagectomy. AQP1 was primarily located in the cytoplasm and/or the nuclear membrane of carcinoma cells. The 5-year survival rate of patients with the “cytoplasm dominant” expression of AQP1 (47.1%) was significantly lower than other patients (83.2%). The depletion of AQP1 using siRNA induced apoptosis in TE5 and TE15 cells. The results of microarray analysis revealed that Death receptor signaling pathway-related genes were changed in AQP1-depleted TE5 cells. In conclusion, the results of the present study suggested that the cytoplasm dominant expression of AQP1 is related to a poor prognosis in patients with ESCC, and that it activates tumor progression by affecting Death receptor signaling pathway. These results provide insights into the role of AQP1 as a mediator of and/or a biomarker for ESCC.

## INTRODUCTION

Esophageal cancer is a malignant tumor with one of the worst prognosis worldwide [1], and squamous cell carcinoma is the predominant histological type of esophageal carcinoma, especially in Eastern countries [2]. Although surgical treatments, adjuvant therapies, and chemoradiotherapies for esophageal cell carcinoma (ESCC) have advanced, treatment outcomes remain challenging, and the 5-year survival rate for advanced cancer remains low because of its highly invasive and metastatic characteristics [1, 2]. To achieve the best possible treatment outcomes of ESCC, a deeper understanding of the molecular mechanisms activating its tumorigenesis and progression is needed.

Aquaporins (AQPs) are transmembrane proteins whose main function is to facilitate the movement of water across cellular membranes, and, therefore, play a major role in body water homeostasis [3]. AQPs also transport other molecules, such as urea and glycerol, and mediate intercellular signals. To date, AQPs have 13 isoforms and their pathophysiological roles in humans have been clarified [4]. For instance, AQP1 is expressed in various tissues, including kidney tubules, endothelia, erythrocytes, choroid plexus, ciliary epithelium, intestinal lacteals, and the corneal endothelium [4].

Recent studies have revealed that AQPs plays crucial roles in various cancers [5–8]. For instance, we have reported previously that AQP5 expression in ESCC cells affects cell proliferation and apoptosis [9]. Qin et al. indicated that AQP1 was localized predominantly in the cytoplasm of cancer cells of invasive breast cancer patients, and that the expression of cytoplasmic AQP1 was an independent prognostic factor [10]. On the other hand, Kang et al. revealed that the expression of AQP1 had no effect on the overall survival rate and disease-free survival rate in patient with colon cancer [11]. However, the expression and pathophysiological roles of AQP1 in human ESCC are still unknown. This research aimed to determine the roles of AQP1 in the control of tumorigenesis-related genes and its clinical meaning in esophageal cancer. By analyzing AQP1 expression in human ESCC tissues, relationships with the clinicopathological features and prognosis of ESCC patients were investigated. In addition, microarray data revealed that knockdown of gene expression using AQP1 siRNA affected a lot of genes related to the Death receptor signaling pathway.

## RESULTS

### Immunohistochemical analysis of AQP1 expression in ESCC tumors

**Figure 1:**
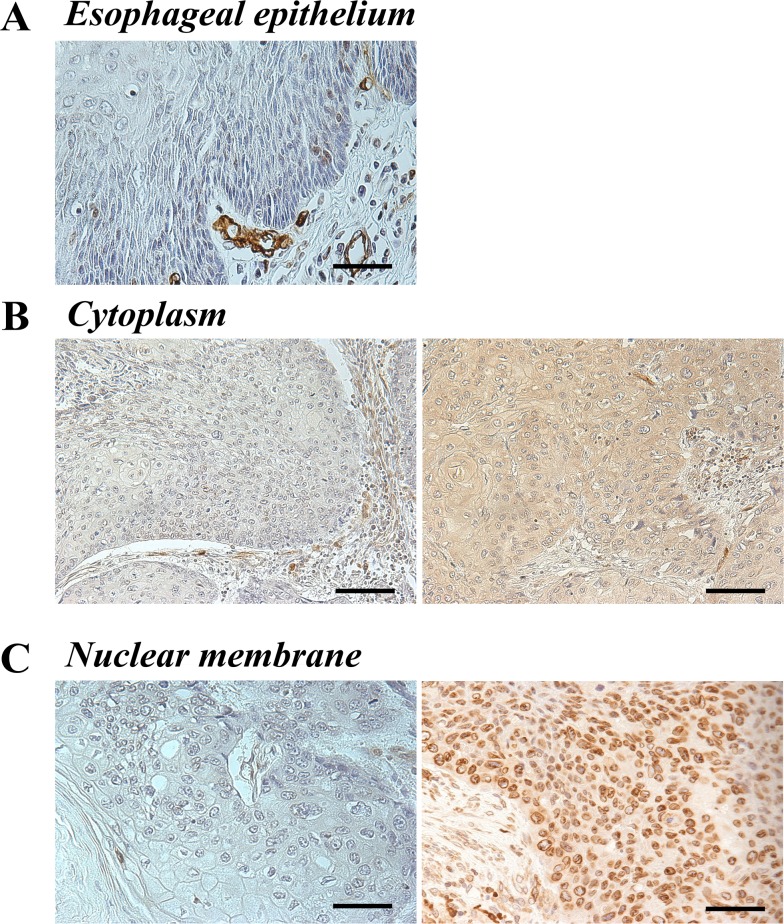
AQP1 protein expression in human ESCC **(A)** Immunohistochemical staining of human esophageal epithelia using an anti-AQP1 antibody. AQP1 was not expressed in noncancerous esophageal squamous epithelium. Magnification: ×400. Bar 50 μm. **(B)** Immunohistochemical staining of the cytoplasm in primary human ESCC samples using an anti-AQP1 antibody. Photomicrographs are shown with the examples of negative cells (*left*), positive cells (*right*). Magnification: ×200. Bar 100 μm. **(C)** Immunohistochemical staining of the nuclear membrane in primary human ESCC samples with an anti-AQP1 antibody. Photomicrographs are shown with the examples of the low AQP1 expression in the nuclear membrane (*left*) and of the high AQP1 expression in the nuclear membrane (*right*). Magnification: ×400. Bar 50 μm.

Immunohistochemistry for the AQP1 protein was performed to investigate the expression of AQP1 in primary tumor tissues of 50 human ESCC samples. It revealed that the expression of AQP1 was localized in the cytoplasm and/or the nuclear membrane of cancer cells, although normal esophageal epithelia did not show staining for AQP1 either in the cytoplasm or the nuclear membrane (Figure 1A).

**Figure 2:**
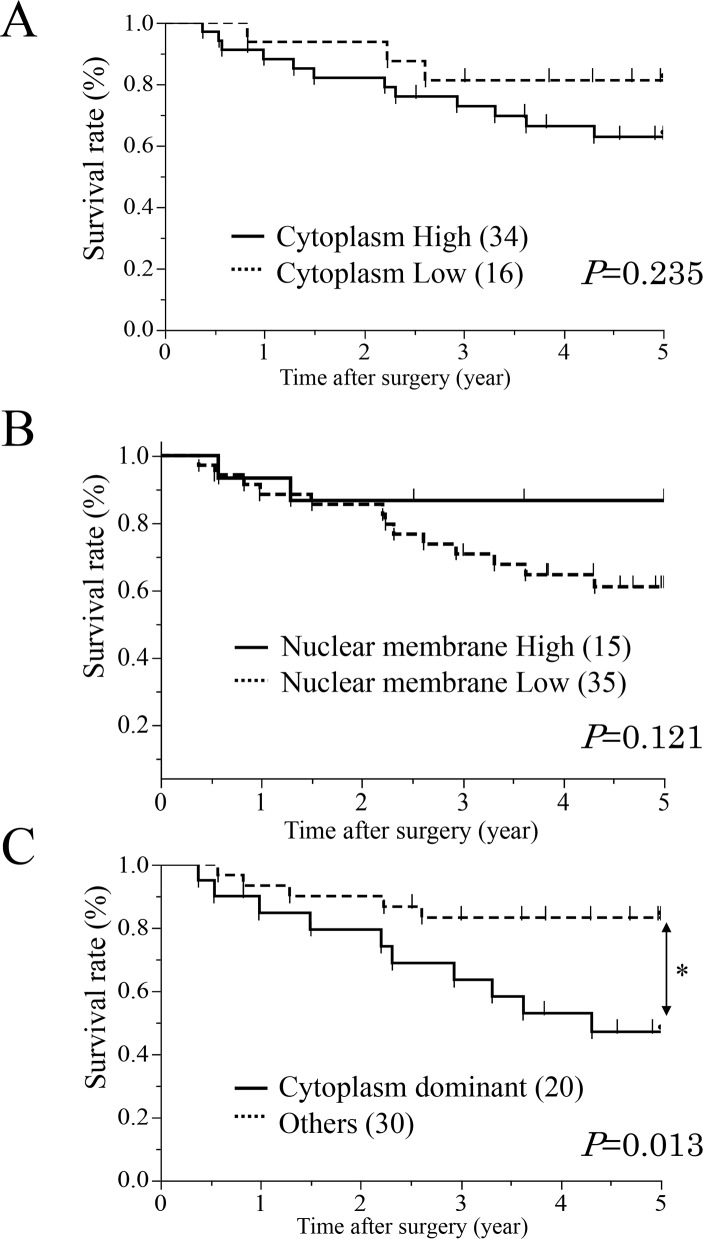
Survival curves of patients after curative resection for ESCC according to the expression of AQP1 **(A)** All patients were classified into two groups “Cytoplasm High/ Cytoplasm Low” according to the expression of cytoplasm: the high group of AQP1 expression in the cytoplasm (n=34, solid line) and the low group of AQP1 expression in the cytoplasm (n=16, doted line). **(B)** All patients were classified into two groups “Nuclear membrane High/ Nuclear membrane Low” according to the expression of nuclear membrane: the high group of AQP1 expression (n=15, solid line) and the low group of AQP1 expression (n=35, doted line) in the nuclear membrane. **(C)** All patients were classified into two groups according to both cytoplasm and nuclear membrane: a “Cytoplasm dominant” group (n=20, solid line) and an Others group (n=30, doted line). ^*^p<0.05: Log-rank test.

First, ESCC patients were categorized into two groups based on expression in the cytoplasm; high (proportion ≥10, n=34) and low (proportion <10, n=16) expression groups (Figure 1B). We investigated the clinicopathological and prognostic significance of AQP1 expression in the cytoplasm after curative resection. The results of clinicopathological analysis showed the AQP1 expression in the cytoplasm did not correlated with any factors. A prognostic analysis showed that the 5-year overall survival rate in the high expression group of the cytoplasm (62.8%) was poorer than that of the low expression group (81.2%), but the difference was not significant (Figure 2A).

Next, we categorized the patients into two groups according to expression in the nuclear membrane; high (proportion ≥30, n=15) and low (proportion <30, n=35) expression groups (Figure 1C). In the analysis of their clinicopathological features, AQP1 expression in the nuclear membrane did not correlate with any features (Table 1A). A prognostic analysis showed that the 5-year overall survival rate in the low expression group in the nuclear membrane (61.0%) was poorer than that of the high expression group (86.6%), but the difference was not significant (Figure 2B).

**Table 1A:** Relationships between clinicopathological features of ESCC and expression of AQP1

	Cytoplasm		*P* value	Nuclear membrane		*P* value
	Low group (n=16)	High group (n=34)		Low group (n=35)	High group (n=15)	
**Sex**						
Male	15	27	0.167	31	11	0.193
Female	1	7		4	4	
**Age**						
<65	8	22	0.324	22	8	0.530
≥65	8	12		13	7	
**Histology type**						
Well/Mod	14	21	0.051	26	9	0.319
Poor	2	13		9	6	
**Location**						
Ce-Ut	1	4	0.670	16	5	0.687
Mt	9	15		16	8	
Lt-Ae	6	15		3	2	
**Tumor size (mm)**						
<50	12	19	0.186	24	7	0.147
≥50	4	15		11	8	
**Lymphatic invasion**						
Negative	5	17	0.208	15	7	0.804
Positive	11	17		20	8	
**Venous invasion**						
Negative	7	19	0.423	18	8	0.902
Positive	9	15		17	7	
**pT**						
pT1	7	12	0.567	13	6	0.848
pT2-4	9	22		22	9	
**pN**						
pN0	9	10	0.070	13	6	0.848
pN1-3	7	24		22	9	

pT: pathological T stage, pN: pathological N stage

^*^ *p*<0.05: Fisher's exact test.

These results indicated that cells showing high expression in the cytoplasm and low expression in the nuclear membrane were associated with worse prognosis. Therefore, we divided patients into two groups based on the expression of AQP1 in the cytoplasm and the nuclear membrane; a “Cytoplasm dominance group” (high expression in the cytoplasm and low expression in the nuclear membrane) and an “Other group”. In the analysis of their clinicopathological features, AQP1 expression of cytoplasm dominance groups correlated with pathological lymph node metastasis stage (*p*=0.028, Table 1B). Additionally, AQP1 expression of cytoplasm dominance groups did not correlated with or without post-operative therapy (*p*=0.468, Table 1B).

**Table 1B:** Relationships between clinicopathological features of ESCC and expression of AQP1

	Other group (n=30)	Cytoplasm dominant group (n=20)	*P* value
**Sex**			
Male	25	17	0.875
Female	5	3	
**Age**			
<65	16	14	0.235
≥65	14	6	
**Histology type**			
Well/Moderate	22	13	0.530
Poor	8	7	
**Location**			
Ce-Ut	3	2	0.621
Mt	16	8	
Lt-Ae	11	10	
**Tumor size (mm)**			
<50	19	12	0.812
≥50	11	8	
**Lymphatic invasion**			
Negative	12	10	0.486
Positive	18	10	
**Venous invasion**			
Negative	14	12	0.354
Positive	16	8	
**pT**			
pT1	13	6	0.338
pT2-4	17	14	
**pN**			
pN0	15	4	0.028^*^
pN1-3	15	16	
**Post-operative therapy**			
None	12	6	0.468
Done	18	14	

pT: pathological T stage, pN: pathological N stage

^*^ *p*<0.05: Fisher's exact test.

A prognostic analysis showed that the 5-year overall survival rate in the cytoplasm dominant group (47.1%) was significantly poorer than that of other group (83.2%) (*p =* 0.013) (Figure 2C, Table 2). We determined which of 9 variables (gender, age, histological degree of the differentiation. of SCC, tumor size, lymphatic invasion, venous invasion, pT and pN categories, and AQP1 expression) influenced prognosis (Table 2). A multivariate analysis of the 5-year overall survival rate, with pT categories, pN categories, lymphatic invasion and venous invasion whose *p*-values were less than 0.300 in the univariate analysis (Table 2), showed that the pT categories, venous invasion and cytoplasm dominance groups of AQP1 were independent prognostic factors (*p =* 0.0423, 0.0473 and 0.0058, respectively) (Table 2).

**Table 2:** Five-year overall survival rate of patients with ECC according to various clinicopathological parameters

	*n*	Univariable		Multivariable		
		5-year OS	P value	Risk Ratio	95% CI	P value
**Sex**						
Male	42	65.2%	0.348			
Female	8	85.7%				
**Age**						
<65	30	65.1%	0.621			
≥65	20	73.7%				
**Tumor size (mm)**						
<50	31	70.0%	0.518			
≥50	19	67.7%				
**Histology type**						
Well/Moderate	35	70.0%	0.701			
Poor	15	65.5%				
**Lymphatic invasion**						
Negative	22	76.2%	0.254	2.685	0.859-9.438	0.0903
Positive	18	62.3%				
**Venous invasion**						
Negative	26	79.3%	0.075	3.174	1.014-11.017	0.0473^#^
Positive	24	56.7%				
**pT**						
pT1	19	78.9%	0.096	3.659	1.044-17.219	0.0423^#^
pT2-4	31	62.5%				
**pN**						
pN0	19	68.4%	0.289	2.167	0.664-8.461	0.2060
pN1-3	31	58.1%				
**AQP1**						
Other	30	83.2%	0.013^*^	4.761	1.567-16.289	0.0058^#^
Cytoplasm dominant	20	47.1%				

pT: pathological T stage, pN: pathological N stage

^*^p < 0.05: Log-rank test.

^#^*p* < 0.05: Cox's proportional hazards model; 95% CI: 95% confidence interval.

### AQP1 protein localization varies depending on ESCC cell lines

**Figure 3:**
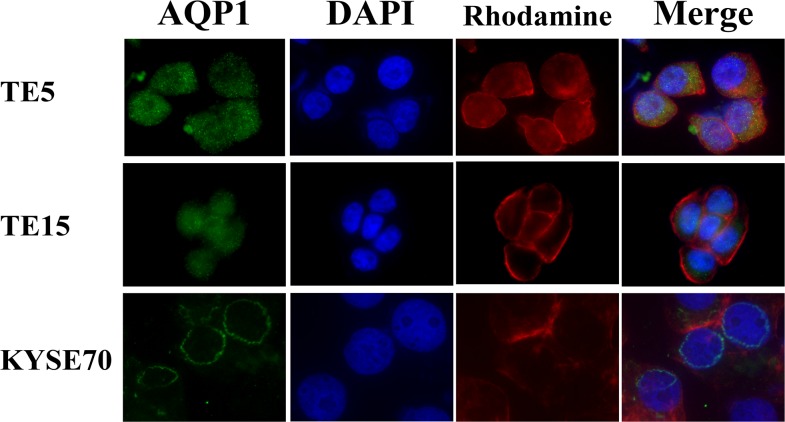
The localization of AQP1 protein differs depending on the type of esophageal cancer cells Immunofluorescent staining of AQP1 on TE5 (*upper*), TE15 (*middle*), and KYSE70 (*lower*) cells. AQP1 protein mainly existed in the cytoplasm of TE5 and TE15 cells, but in the nuclear membrane of KYSE70 cells.

According to the result of immunohistochemistry, we hypothesized that tumor cells possessed different types of AQP1 phenotype in ESCC tissues and that it may affect the prognosis of esophageal cancer. Therefore, we investigated the location of AQP1 protein in TE5, TE15, and KYSE70 cells using immunofluorescence analysis. In order to recognize the localization of AQP1 more clearly, the cytoskeleton was labeled with Rhodamine and the nuclear was labeled with DAPI. In TE5 and TE15 cells, AQP1 protein mainly existed in the cytoplasm (Figure 3). On the other hand, the expression of AQP1 in KYSE170 cells was confirmed on the nuclear membrane (Figure 3). These findings of immunofluorescence were consistent with our analysis of immunohistochemistry.

### AQP1 suppresses apoptosis in ESCC cells

**Figure 4:**
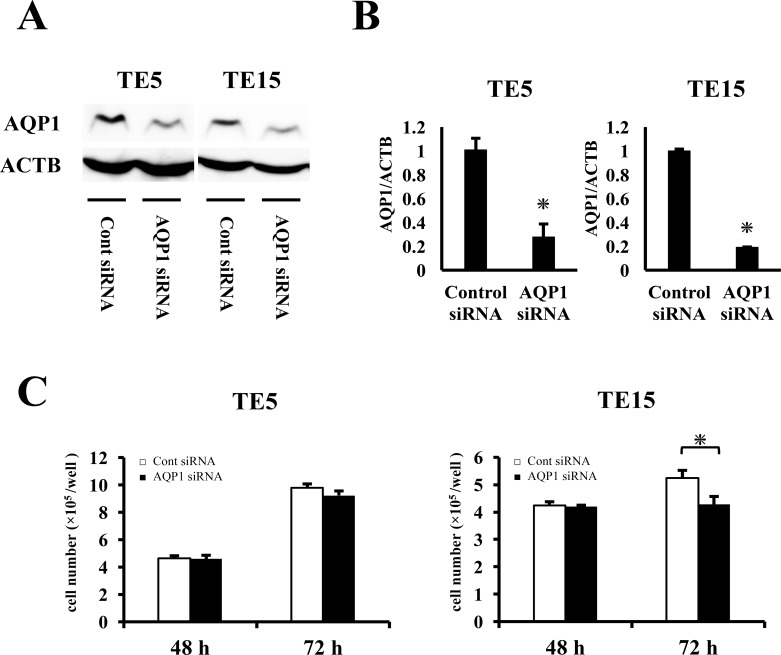
The proliferation with AQP1-depleted *TE5 and TE15 cells* **(A)** Western blotting revealed that AQP1 siRNA effectively reduced AQP1 protein levels in TE5 and TE15 cells. **(B)** AQP1 siRNA effectively reduced AQP1 mRNA levels in TE5 and TE15 cells. Mean ± SEM. n = 3. ^*^p < 0.05 (significantly different from control siRNA). **(C)** The down-regulation of AQP1 inhibited the proliferation of TE5 and TE15 cells. The number of cells was counted 48 and 72 h after siRNA transfection. Mean ± SEM. n = 3. ^*^p < 0.05 (significantly different from control siRNA).

**Figure 5:**
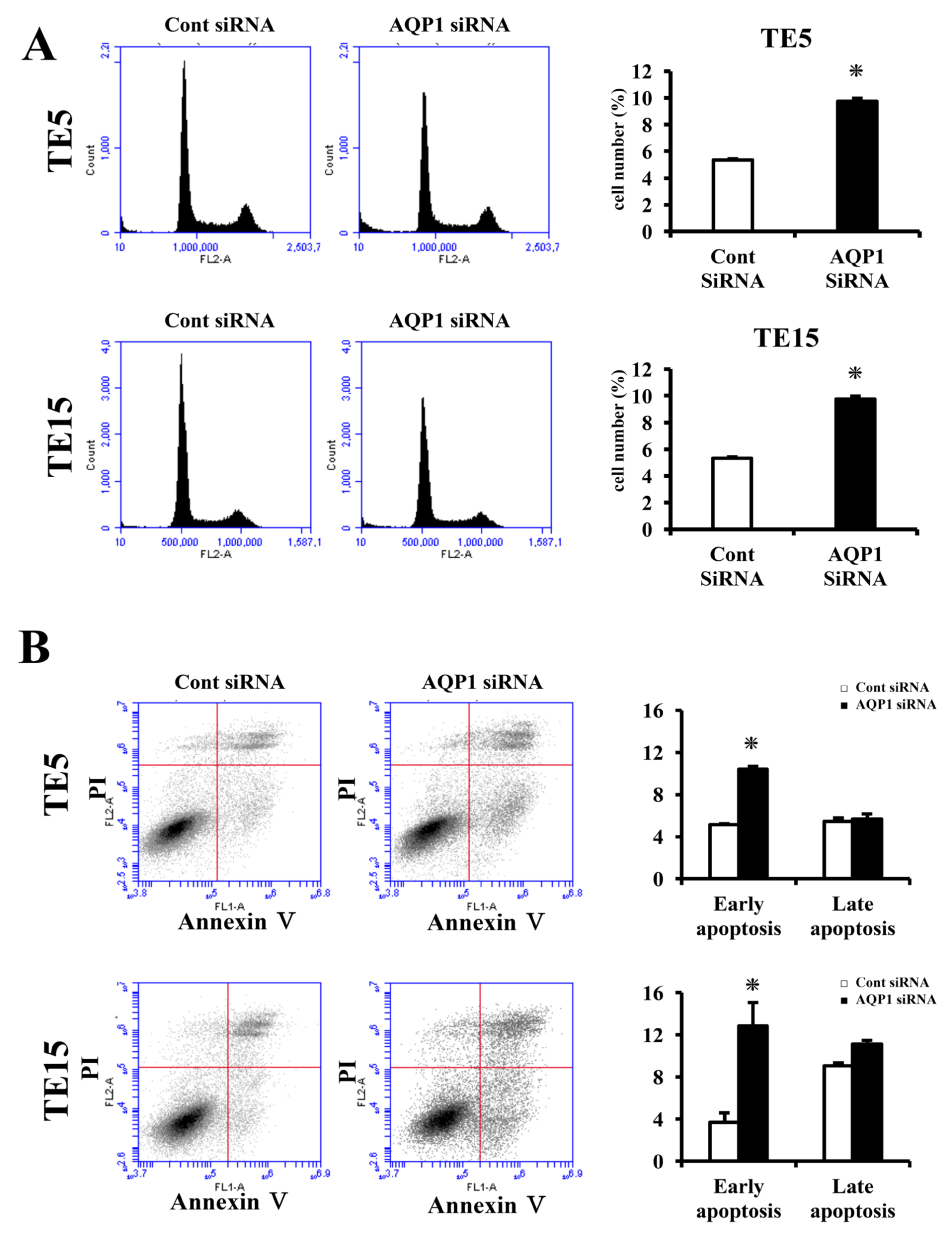
AQP1 suppress apoptosis in ESCC cells **(A)** Down-regulation of AQP1 increases the component of cells in subG1 phase of TE5 and TE15 cells. Cells transfected with control or AQP1 siRNA were stained with propidium iodide (PI) and analyzed by flow cytometry. Mean ± SEM. n = 3. ^*^p < 0.05 (significantly different from control siRNA). **(B)** AQP1 had influence on apoptosis in TE5 and TE15 cells. Apoptosis was determined by flow cytometry using PI/Annexin V double staining. Mean ± SEM. n = 3. ^*^p < 0.05 (significantly different from control siRNA).

In order to the elucidate functions of AQP1 in ESCC, we performed knockdown experimentations using AQP1 siRNA in TE5 and TE15 cell lines and investigated influences on cell proliferation and the cell cycle. AQP1 protein and mRNA levels were obviously decreased by AQP1 siRNA transfection in both cell lines (Figure 4A, 4B). TE15 cell counts 72 h after siRNA transfection were significantly lower in AQP1 siRNA-transfected cells than in control cells (Figure 4C). In TE5 cells, cell proliferation was lower in AQP1 siRNA-transfected cells than in control cells (Figure 4C). Furthermore, the knockdown of AQP1 significantly increased the component of subG1 phase in the cell cycle of both TE5 and TE15 cells (Figure 5A). According to these outcomes, we hypothesized that cells with depleted AQP1 were induced to undergo apoptosis.

Next, we transfected TE5, TE15, and KYSE70 cells with AQP1 siRNA and examined apoptosis. AQP1 depletion significantly increased early apoptosis (Annexin V positive/PI negative) in TE5 and TE15 cell lines at 72 h after siRNA transfection (Figure 5B). In contrast, the down-regulation of AQP1 did not increase early apoptosis in KYSE70 cells (Supplementary Figure 1). These findings indicated that the expression of AQP1 suppresses apoptosis according to the type of ESCC cells, especially where AQP1 expression was predominantly in the cytoplasm. These results supported our hypothesis.

### The migration and invasion assay with AQP1-depleted TE5 and TE15 cells

**Figure 6:**
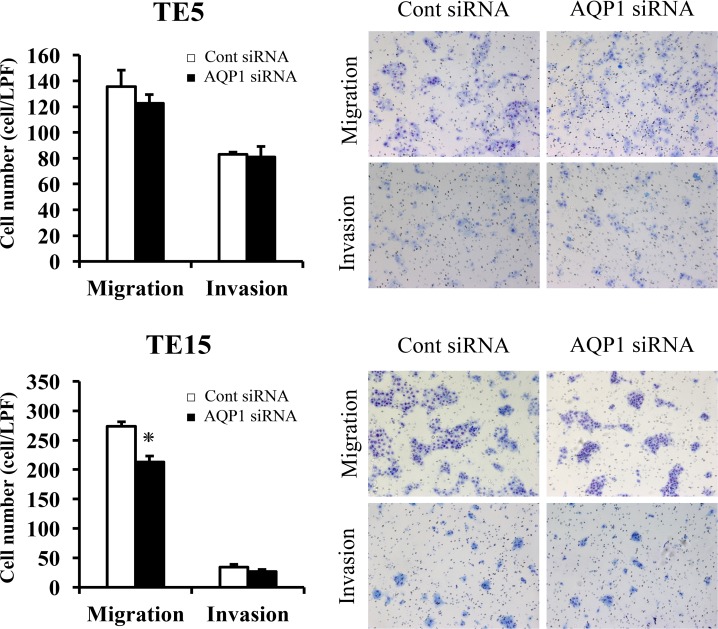
The migration and invasion assay with *AQP1*-depleted TE5 and TE15 cells The down-regulation of AQP1 inhibited the migration of TE15 cells but did not inhibit the invasion of TE5 or TE15 cells. Cell migration and invasion were examined using the Boyden chamber assay. Mean ± SEM. n = 3. ^*^p < 0.05 (significantly different from control siRNA).

In TE15 cells, AQP1 siRNA significantly reduced cell migration (Figure 6). In TE5 and TE15 cells, AQP1 depletion did not reduced cell invasion (Figure 6). Previous studies reported that AQP1 also has a role of cell migration and invasion in various cells, including cancer cells [12, 13]. These findings indicated that AQP1 has different capabilities for cell migration and invasion among types of esophageal cancer cells.

### Gene expression profiling in AQP1 siRNA-transfected cells

To determine the molecular mechanisms by which AQP1 regulates cellular functions, we analyzed the gene expression profiles of AQP1-depleted TE5 cells using microarray and bioinformatic studies. The results of the microarray analysis showed that the expression levels of 5000 genes displayed fold changes of > 1.4 in TE5 cells after the depletion of AQP1. Of these genes, 1946 were upregulated and 3054 were downregulated in AQP1 siRNA-depleted TE5 cells. A list of 20 genes with expression levels that were the most strongly up- or downregulated in AQP1-depleted TE5 cells is shown in Supplementary Table 1. An ingenuity pathway analysis (IPA) showed that “Cancer” was the top-ranked disease and that “Cellular Movement”, “Cellular Development”, and “Cellular Growth and Proliferation” were some of the top-ranked biological functions related to the depletion of AQP1 (Supplementary Table 2).

### Verification of gene expression by real-time quantitative RT-PCR and western blotting

**Figure 7:**
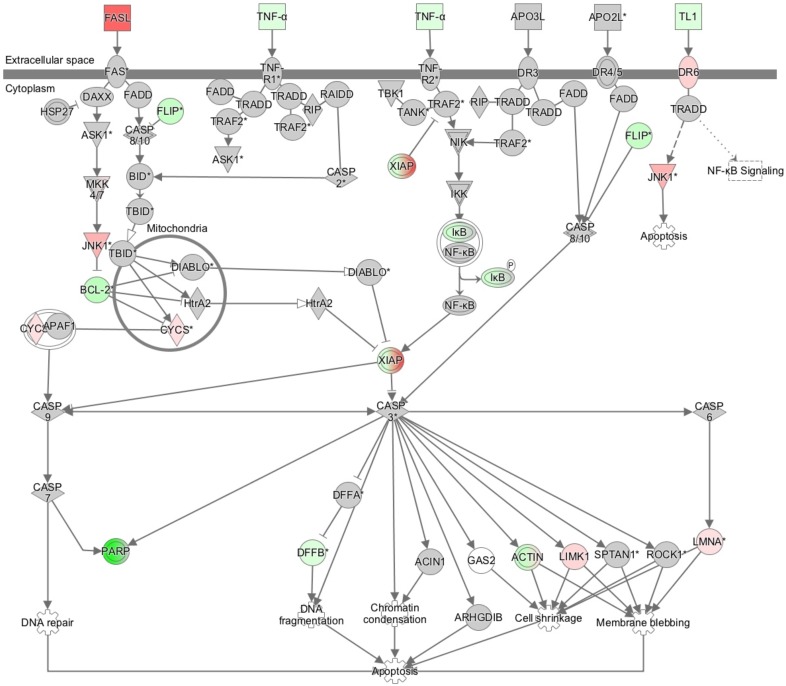
The signaling map of “death receptor signaling”, showing the canonical pathways related to AQP1 depletion according to an Ingenuity Pathway Analysis Red and green indicate genes with expression levels that were higher or lower, respectively, than reference RNA levels.

**Figure 8:**
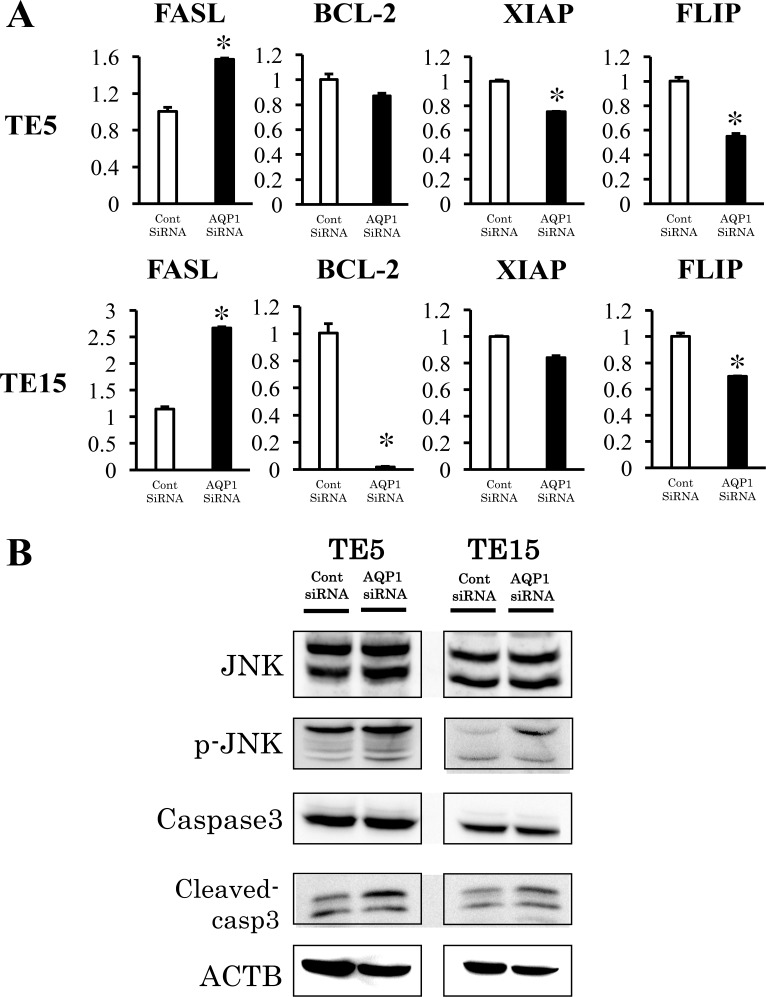
Signaling pathways regulated by AQP1 in ESCC cells **(A)** Verification of gene expression by real-time quantitative RT-PCR. The expression levels of four selected Death receptor signaling pathway-related genes (FASL, BCL-2, XIAP, and FLIP) in AQP1- depleted TE5 and TE15 cells were compared to those in control siRNA-transfected cells using real-time quantitative RT-PCR. Mean ± SEM. n = 3. ^*^p < 0.05 (significantly different from control siRNA). **(B)** The down-regulation of AQP1 increased the phosphorylation levels of JNK and the levels of Cleaved-caspase 3 in TE5 and TE15 cells.

The results of the microarray analysis also indicated that Death receptor signaling was upregulated by the knockdown of AQP1. (Figure 7, Table 3). We selected four genes (FASL, BCL-2, FLIP, and XIAP) to confirm the results of the microarray analysis. These genes were included in Death receptor signaling. The expression of the four genes was examined using quantitative RT-PCR. The expression level of FASL was significantly higher and that of FLIP was significantly lower in AQP1-depleted TE5 and TE15 cells than in control siRNA-transfected cells (Figure 8A). A western blotting analysis revealed that the down-regulation of AQP1 increased the phosphorylation levels of JNK and cleaved Caspase 3 in TE5 and TE15 cells (Figure 8B). These results were consistent with the microarray results and suggested that knockdown of AQP1 suppresses Death receptor signaling in ESCC cells.

**Table 3:** Death receptor signaling pathway-related genes with expression levels in TE5 cells that were changed by the depletion of AQP1

Death receptor signaling			
Symbol	Gene Name	UniGene ID	Expr Fold Change
FASLG	Fas ligand	Hs.2007	7.204
MAPK8	mitogen-activated protein kinase 8	Hs.138211	2.642
CYCS	cytochrome c, somatic	Hs.437060	1.486
LMNA	lamin A/C	Hs.594444	1.43
BCL2	BCL2, apoptosis regulator	Hs.150749	−2.648
CFLAR	CASP8 and FADD like apoptosis regulator	Hs.390736	− 2.336
XIAP	X-linked inhibitor of apoptosis	Hs.356076	− 1.379

## DISCUSSION

Aquaporins (AQPs) which have 13 isoforms, are transmembrane proteins whose main function is to facilitate the movement of water across cellular membranes. AQPs play a major role in body water homeostasis, such as epithelial secretion, absorption, and cell volume regulation [3, 14]. Recent researches revealed that AQP1, one of AQPs, also were implicated in localized protrusions of plasma membranes, cell motility, and angiogenesis [13, 15]. Further, recent studies using immunohistochemical examinations have shown that AQPs expression in various human carcinoma tissues correlated with prognosis [10, 11, 16–19]. Several reports have revealed the expression and roles of AQPs in human ESCC, such as AQP3, 4, 5, and 8 [9, 20–23]. We also previously demonstrated that the overexpression of AQP5 in ESCC promoted cell proliferation and suppressed apoptosis [9], as we have been researching the expression of channels/transporters and their roles in ESCC cells [9, 24–28]. Regarding the expression of AQP1, Kao et al. revealed that the expression of AQP1 ≥50% in malignant mesothelioma cells was an independent factor of poor prognosis [16]. Furthermore, in the present study, we investigated the correlation between AQP1 expression in ESCC and clinicopathological factors and prognoses. Our results showed that AQP1 expression of cytoplasm dominance groups correlated with pathological lymph node metastasis stage (*p*=0.028, Table 1B). Yoshida et al also revealed that the AQP1 expression exhibited a significant correlation with lymph node metastasis, severe lymphovascular invasion and vascular invasion in colon cancer [18]. Although the reason why the AQP1 expression of cytoplasm dominance groups was only correlated with pN categories is still unrevealed, further investigation may afford a deeper understanding of this mechanism. Our results revealed that 5-year overall survival rate of the cytoplasm dominant group of AQP1 was significantly poorer than those of other group of AQP1. On the other hand, our results suggested that ESCC cells in which AQP1 is expressed predominantly in the cytoplasm and plays a crucial role in tumor progression.

Previous reports showed that the expression of AQP1 was overexpressed in lung adenocarcinoma [29–31]. Lehnerdt et al. reported that AQP1 was strongly expressed in pharyngeal basaloid-type SCC in pharyngeal SCC [32]. Our data also showed that AQP1 was highly expressed in esophageal adenocarcinoma (Supplementary Figure 2A) and in esophageal basaloid-type SCC (Supplementary Figure 2B). Further, our immnohistochemical analysis indicated that AQP1 was expressed in esophageal squamous cell carcinoma and affect poor prognosis.

In our study, we need to mention the limitations of this retrospective study. Certainly, the factors of pN and pT categories were not significantly correlated with 5-year overall survival rate, but they were tended to be correlated with it. We considered that one of the reasons was related to small sample size in our study. Recently preoperative therapy has been performed positively for advanced ESCC in Japan [33], and therefore, it has become difficult to obtain samples without neoadjuvant therapy from patient with advanced ESCC.

Regarding the role of AQP1 in cancer cells, previous studies have shown that the expression of AQP1 is correlated with cell proliferation, migration, and angiogenesis in several cancers [13–15, 17, 34–36]. For instance, Wei et al. showed that the proliferation of depleted-AQP1 lung adenocarcinoma cells was significantly inhibited [34]. Saadoun et al. indicated that targeted AQP1 gene disruption of melanoma cells reduced angiogenesis *in vivo* [13]. Furthermore, Monzani et al. reported that AQP1 played a role in cell migration according to interacting with Lin-7/Δ-catenin in human melanoma cells [15]. In the present study, cell cycle analysis indicated that the knockdown of AQP1 with siRNA increased the component of sub G1 phase in TE5 and TE15, ESCC cell lines. Furthermore, apoptosis analysis revealed that depleted-AQP1 ESCC cells were induced to undergo apoptosis. These findings indicated that AQP1 expression may suppress apoptosis.

Recent reports using immunofluorescence analyses have shown that the localization of AQP1 was in the cell surface membrane and/or the cytoplasm in various cancer cells [14, 15, 34]. Our immunohistochemical analysis revealed that the localization of AQP1 expression differed among samples in ESCC. Further we showed that 5-year overall survival rate with high AQP1 expression in the cytoplasm was lower than with low, and that 5-year overall survival rate with low AQP1 expression in the nuclear membrane was lower than with high (Figure 2A, 2B). We indicated with immunofluorescence analysis that AQP1 was present predominantly in the cytoplasm in TE5 and TE15 cells, although AQP1 was present in the nuclear membrane in KYSE70 cells (Figure 3). *In vitro* experiments, we demonstrated that AQP1-depleted TE5 and TE15 cells, which AQP1 predominantly expressed in the cytoplasm was accelerated apoptosis (Figure 5B), although apoptosis was not increased in AQP1-depleted KYSE70 cells, which AQP1 predominantly expressed in the nuclear membrane. (Supplementary Figure 1B). Previous studies including our report [37] revealed that several molecules, such as E-cadherin, Δ-catenin, ZO-1, ZO-2, and claudin-1 affected tumor progression, according to the change in their own intracellular localization [38]. Although the mechanisms in which localization varies depending on ESCC cells are still unknown and require further investigation, the results of immunofluorescence analysis support our IHC analysis in which ESCC cells expressing AQP1 predominantly in the cytoplasm are involved in tumor progression.

Death receptor signaling is known to activate caspase-induced apoptosis [39–42]. Apoptosis is induced by two mechanisms: the extrinsic pathway associated with Death receptor stimulation on the cell surface, and the intrinsic pathway characterized by the involvement of mitochondrial dysfunction. Death receptors belong to the tumor necrosis factor receptor superfamily, including Fas (CD95/APO-1), TNF-R1, TNF-related apoptosis-inducing ligand (TRAIL)-receptor 1 (TRAIL-R1, DR4), and TRAIL-R2 (APO-2, DR5). Fas, one of the Death receptors, is induced by oligomerization by binding with the Fas-ligand, and this binding triggers the formation of a pro-apoptotic protein complex termed the Death inducing signaling complex (DISC) composed of FADD (Fas-associated death domain protein) and caspase-8. As a result of these interactions, caspase8 is activated and a proteolytic caspase cascade is triggered. Active caspase-8 induced directly the activation of caspase-3 and/or activate themitochondrial apoptosis pathway via the cleavage of Bid protein [40]. Furthermore, previous reports revealed that Fas receptor induces apoptosis via activation of JNK [43]. On the other hand, this signaling pathway is suppressed at each step by inhibitory proteins, such as decoy receptors (in the case of TRAIL signaling), cFLIP isoforms [44], anti-apoptotic Bcl2 family members, and IAP family proteins [45]. The results of the present study indicated the gene expression of factors that activate the Death receptor signaling pathway, such as FASL, cleaved caspase-3, FLIP, XIAP, and p-JNK was changed by the knockdown of AQP1, suggesting that AQP1 suppresses this pathway in ESCC cells.

In summary, we have shown that AQP1 plays a role in suppressing apoptosis in ESCC cells lines and that the cytoplasmic dominant AQP1 expression was a prognostic factor in human ESCC tissues with immunohistochemically detected expression. Our microarray data also indicated that AQP1 affects the expression of genes with functions related to cellular movement, growth, and proliferation and gene expressions associated with the Death receptor signaling pathway. Although further investigations of the molecular mechanism are required, our observations suggested that AQP1 may be one of the key biomarkers. A deeper understanding of AQP1 mechanisms in cancer cells could lead to the development of novel therapeutic strategies in ESCC.

## MATERIALS AND METHODS

### Cell culture, antibodies, and other materials

Human ESCC cell lines TE5 (poorly differentiated type) and TE15 (well differentiated type) were obtained from the Cell Resource Center for Biomedical Research at the Institute of Development, Aging, and Cancer (Tohoku University, Sendai, Japan). Human ESCC cell line KYSE70 (poorly differentiated type) was obtained from the Japanese Collection of Research Bioresources Cell Bank (Osaka, Japan). These cell lines were grown in RPMI-1640 medium (Nacalai Tesque, Kyoto, Japan) supplemented with 100 U/mL penicillin, 100 μg/mL streptomycin, and 10% fetal bovine serum (FBS). Cells were cultured in flasks and dishes in a humidified incubator at 37°C in 5% CO_2_ in air. The monoclonal anti-AQP1 antibody used for the immunohistochemical analysis, immunofluorescence analysis, and protein assay was obtained from Santa Cruz Biotechnology (Santa Cruz, CA, USA). The following antibodies were used in the western blotting analysis; rabbit polyclonal anti-Jun-amino-terminal kinase (JNK) antibody, rabbit monoclonal anti-phosphoJNK antibody, rabbit monoclonal anti-Caspase 3 antibody, and rabbit monoclonal anti-Cleaved-Caspase 3 antibody were purchased from Cell Signaling Technology (Beverly, MA). A mouse monoclonal anti-β-actin antibody was purchased from Sigma-Aldrich (St. Louis, MO, USA).

### Western blotting

Cells were harvested in M-PER lysis buffer (Pierce, Rockford, IL) supplemented with protease inhibitors (Pierce, Rockford, IL). Protein concentrations were measured with a modified Bradford assay (Bio-Rad, Hercules, CA). Cell lysates containing equal amounts of total protein were separated by SDS-PAGE and then transferred onto PVDF membranes (GE Healthcare, Piscataway, NJ). These membranes were then probed with the indicated antibodies, and proteins were detected using an ECL Plus Western Blotting Detection System (GE Healthcare, Piscataway, NJ).

### Small interfering RNA (siRNA) transfection

Cells were transfected with 20 nmol/L AQP1 siRNA (Stealth RNAi siRNA #HSS141260, Invitrogen, Carlsbad, CA) using Lipofectamine RNAiMAX reagent (Invitrogen) in accordance with the manufacturer's instructions. Medium containing siRNA was replaced with fresh medium after 24 h. Control siRNA (Stealth RNAi siRNA Negative Control; Invitrogen) was used as a negative control.

### Cell proliferation

Cells were seeded on 6-well plates at a density of 1.2 × 10^5^ cells per well for TE5 and 1.5 × 10^5^ cells per well for TE15, and incubated at 37°C with 5% CO_2_. siRNA was transfected 24 h after the cells had been seeded. Cells were detached from the flasks with trypsin-EDTA 48 and 72 h after siRNA transfection and were counted using a hemocytometer.

### Analysis of apoptotic cells

Cells were harvested 72 h after siRNA transfection and stained with fluorescein isothiocyanate (FITC)-conjugated Annexin V and propidium iodide (PI) using an Annexin V-FITC kit (Beckman Coulter, Brea, CA) in accordance with the manufacturer's protocol. The proportion of apoptotic cells was analyzed by fluorescence-activated cell scoring (FACS) using a BD Accuri C6 (BD Biosciences).

### Real-time reverse transcription-polymerase chain reaction (RT-PCR)

Total RNA was extracted using an RNeasy kit (Qiagen, Valencia, CA). mRNA expression levels were measured by quantitative real-time PCR (7300Real-Time PCR System; Applied Biosystems, Foster City, CA) using TaqMan Gene Expression Assays (Applied Biosystems) in accordance with the manufacturer's instructions. Expression levels were measured for the following genes: AQP1 (Hs01028916_m1), FASL (Hs00181225_m1), XIAP (Hs00745222_s1), FLIP (Hs01117851_m1), and BCL-2 (Hs00608023_m1) (Applied Biosystems). For the AQP1 gene, expression was normalized to the housekeeping gene beta-actin (ACTB, Hs01060665 g1; Applied Biosystems). Assays were performed in duplicate.

### Cell cycle analysis

In AQP1 knockdown experiments, cell cycle progression was evaluated 48 h after siRNA transfection using FACS. In heat shock experiments, cell cycle progression was evaluated 24 h after the heat shock treatment for 2 h. Briefly, cells were treated with Triton X-100, and cell nuclei were stained with PI RNase staining buffer (Becton-Dickinson Biosciences, San Jose, CA, USA). The DNA content was then measured using a Becton-Dickinson Accuri C6 (Becton-Dickinson Biosciences). At least 10,000 cells were counted, and BD Accuri C6 software was used to analyze the cell cycle distribution.

### Analysis of cell migration and invasion

The migration assay was conducted using a Cell Culture Insert with a pore size of 8 μm (BD Biosciences, Bedford, MA, USA). Biocoat Matrigel (BD Biosciences) was used to evaluate cell invasion potential. Cells (TE5: 3 × 10^5^ cells per well/ TE5: 6 × 10^5^ cells per well), were seeded in the upper chamber in serum-free medium 24 h after siRNA transfection. The lower chamber contained medium with 10% FBS. The chambers were incubated at 37°C for 48 h in 5% CO_2_, and non-migrated or non-invaded cells were then removed from the upper side of the membrane by scrubbing with cotton swabs. Migrated or invaded cells were fixed on the membrane and stained with Diff-Quick staining reagents (Sysmex, Kobe, Japan). The migrated or invaded cells on the lower side of the membrane were counted in four independent fields of view at 100× magnification for each insert. Each assay was performed in triplicate.

### Microarray sample preparation and hybridization

Total RNA was extracted using an RNeasy kit (Qiagen). RNA quality was monitored with an Agilent 2100 Bioanalyzer (Agilent Technologies, Santa Clara, CA). Cyanine-3 (Cy3)-labeled cRNA was prepared from 0.1 μg of total RNA using a Low Input Quick Amp Labeling Kit (Agilent) in accordance with the manufacturer's instructions. Samples were purified using RNeasy columns (Qiagen). A total of 0.60 μg of Cy3-labeled RNA was fragmented and hybridized to an Agilent SurePrintG3 Human Gene Expression 8 × 60 K ver2.0 Microarray for 17 h. Slides were washed and scanned immediately using an Agilent DNA Microarray Scanner (G2565CA) in the one color scan setting for 8 × 60 K array slides.

### Processing of microarray data

Scanned images were analyzed using Feature Extraction Software 10.10 (Agilent) using default parameters to obtain background-subtracted and spatially detrended Processed Signal intensities. Signal transduction networks were analyzed using an Ingenuity Pathway Analysis (IPA) (Ingenuity Systems, Qiagen, Redwood City, CA).

### Patients and primary tissue samples

ESCC tumor samples were obtained from 50 patients with histologically confirmed primary ESCC who underwent esophagectomy at Kyoto Prefectural University of Medicine between 1999 and 2009 and were embedded in paraffin after 12 h of formalin fixation. Patient eligibility criteria were as follows: no synchronous or metachronous cancers (in addition to ESCC) and no preoperative chemotherapy or radiation therapy. We excluded patients with non-curative resected tumors. All patients provided written informed consent. Relevant clinicopathological and survival data were obtained from the hospital database, and we showed these detailed backgrounds of 50 patients in Supplementary Table 3. Staging was principally based on the International Union Against Cancer/tumor node metastasis Classification of Malignant Tumors (7th edition).

### Immunohistochemistry

Paraffin sections (4 μm thick) of tumor tissues were subjected to immunohistochemical staining using the avidin-biotin-peroxidase method. Briefly, paraffin sections were dewaxed with xylene and hydrated with a graded series of alcohol. Endogenous peroxidases were quenched by incubating the sections for 30 min in 0.3% H_2_O_2_. For blocking of endogenous biotin, biotin receptors, and avidin binding sites, Avidin/Biotin Blocking Kit was used (Vector Laboratories, Burlingame, CA). Sections were then treated with a protein blocker and incubated at 4°C overnight with the primary antibody. The avidin-biotin-peroxidase complex (Vectastain ABC Elite kit; Vector Laboratories, Burlingame, CA) was visualized using diaminobenzidine tetrahydrochloride. Sections were counterstained with hematoxylin. These sections were then dehydrated through a graded series of alcohols, cleared in xylene, and mounted.

Immunohistochemical samples stained with AQP1 were graded semi-quantitatively based on the staining intensity and percentage of positive tumor cells. First, tumor cells were divided into negative and positive groups based on the staining intensity of AQP1 in the cytoplasm, and the proportion of positive tumor cells in the cytoplasm was scored from 0 to 100. Therefore, patients were categorized into two groups based on the proportion of expression in the cytoplasm (range=0–80, mean±SE=24.4±3.62); high (proportion ≥10, n=34) and low (proportion <10, n=16) expression groups (Figure 1B). Next, tumor cells were also divided into negative and positive groups based on the staining intensity of AQP1 in the nuclear membrane. The proportion of stained tumor cells in the nuclear membrane was scored from 0 to 100. As with the categorization of the cytoplasm, we divided patients into two groups based on the proportion of the expression in the nuclear membrane (range=0–80, mean±SE=18.8±3.28); high (proportion ≥30, n=15) and low (proportion <30, n=35) expression groups (Figure 1C). We measured the proportion of positive cells in the whole tumor tissue with phase contrast microscope at 100 magnification, and decided the expression proportion of cytoplasm and nuclear membrane. The cut off value of patients divided into two groups for cytoplasm and nuclear membrane respectively was determined as the value which 5-year overall survival rate between two groups was the most difference. We showed 5-year overall survival rate with each cut-off values in Supplementary Table 4.

### Immunofluorescence staining

Cells were stained in accordance with a standard cell staining protocol. Briefly, TE5, TE15, and KYSE70 cells were cultured on SPL cell culture slides, which are 8-chamber slides (SPL Life Science, Pocheon, Korea) for 24 h. Cells were subsequently fixed with 4% paraformaldehyde at room temperature for 20 min, permeabilized in 0.1% Triton X-100 in phosphate-buffered saline (PBS) for 1 min, and incubated in blocking buffer containing 1% bovine serum albumin for 30 min. Cells were then incubated with the anti-AQP1 antibody at room temperature overnight. After three washes in PBS, cells were incubated with Alexa Fluor 488-labeled goat anti-mouse secondary antibodies at room temperature for 1 h. After three washes in PBS, cells were incubated with rhodamine phalloidin and 40,6-diamidino-2-phenylindole (DAPI) for 30 min. Slides were then mounted with Vectashield Mounting Medium (Vector Laboratories, Burlingame, CA, USA). The distribution of AQP1 proteins was examined using a BZ-X700 (Keyence, Tokyo, Japan).

### Statistical analysis

Fisher's exact test was used to evaluate differences between proportions, and the Student's *t*-test was employed to evaluate continuous variables. Survival curves were constructed using the Kaplan–Meier method, and differences in survival were examined using the Log-rank test. A multivariate analysis of the factors influencing survival was performed using a Cox's proportional hazard model. Differences were considered significant when the relevant *p* value was <0.05. These analyses were performed using JMP statistical software (version 12, SAS Institute Inc., Cary, NC).

## SUPPLEMENTARY MATERIALS FIGURES AND TABLES


