# Supplementary material for: Aquaporin 1 suppresses apoptosis and affects prognosis in esophageal squamous cell carcinoma
Source: Oncotarget. 2018 Jul 6;9(52):29957–74. doi: 10.18632/oncotarget.25722 (PMC6057448; doi:10.18632/oncotarget.25722)
Supplement: Supplementary file 1 [file oncotarget-09-29957-s001.pdf]

Aquaporin 1 suppresses apoptosis and affects prognosis in esophageal squamous cell carcinoma

SUPPLEMENTARY MATERIALS

Supplementary Table 1A: The 20 genes that displayed the most up-regulated change in their expression levels in the AQP1 siRNA transfected TE5 cells

Symbol	Gene Name	UniGene ID	Expr Fold Change
TRPC7	Transient receptor potential cation channel subfamily C member 7	Hs.591263	176.947
FCRL3	Fc receptor like 3	Hs.292449	128.186
GPC5	Glypican 5	Hs.655675	100.456
OR6C1	Olfactory receptor family 6 subfamily C member 1	Hs.553763	83.208
FGF1	Fibroblast growth factor 1	Hs.483635	79.713
CBR1	Carbonyl reductase 1	Hs.88778	79.535
BOD1L2	Biorientation of chromosomes in cell division 1 like 2	Hs.127882	73.096
THPO	Thrombopoietin	Hs.1166	65.493
IRF7	Interferon regulatory factor 7	Hs.166120	58.113
MYOT	Myotilin	Hs.84665	56.299
GABBR1	Gamma-aminobutyric acid type B receptor subunit 1	Hs.167017	51.456
NXF5	nuclear RNA export factor 5	Hs.307077	46.64
GSTM2	Glutathione S-transferase mu 2	Hs.279837	44.351
NME9	NME/NM23 family member 9	Hs.660992	42.84
MS4A4A	Membrane spanning 4-domains A4A	Hs.325960	42.187
TMEM132B	Transmembrane protein 132B	Hs.524838	42.053
TAS2R13	Taste 2 receptor member 13	Hs.679407	39.57
ETFBKMT	Electron transfer flavoprotein beta subunit lysine methyltransferase	Hs.740628	38.782
SERPINC1	Serpin family C member 1	Hs.75599	37.255
ABCA8	ATP binding cassette subfamily A member 8	Hs.58351	34.346

Supplementary Table 1B: The 20 genes that displayed the most down-regulated change in their expression levels in the AQP1 siRNA transfected TE5 cells

Symbol	Gene Name	UniGene ID	Expr Fold Change
PKD2L2	Polycystin 2 like 2, transient receptor potential cation channel	Hs.716884	-138.177
FAM196B	Family with sequence similarity 196 member B	Hs.721917	-127.583
MCMDC2	Minichromosome maintenance domain containing 2	Hs.677620	-100.672
CCR2	C-C motif chemokine receptor 2	Hs.132310	-100.025
ABCB5	ATP binding cassette subfamily B member 5	Hs.437006	-94.806
BARHL2	BarH like homeobox 2	Hs.511794	-93.257
MAGEE2	MAGE family member E2	Hs.404102	-93.173
OSBPL6	Oxysterol binding protein like 6	Hs.451956	-84.326
AATK	Apoptosis associated tyrosine kinase	Hs.356869	-82.49
TEX12	Testis expressed 12	Hs.318775	-82.283
NDNF	Neuron derived neurotrophic factor	Hs.514575	-80.469
CSDC2	Cold shock domain containing C2	Hs.524039	-76.808
PARP15	Poly(ADP-ribose) polymerase family member 15	Hs.709520	-73.573
PROK2	Prokineticin 2	Hs.310893	-73.559
CPO	Carboxypeptidase O	Hs.120250	-72.393
CCDC127	Coiled-coil domain containing 127	Hs.528665	-71.182
TTLL9	Tubulin tyrosine ligase like 9	Hs.684103	-70.434
MEOX1	Mesenchyme homeobox 1	Hs.293833	-69.256
COL13A1	Collagen type XIII alpha 1 chain	Hs.712915	-66.308
OR10Q1	Olfactory receptor family 10 subfamily Q	Hs.438	-65.996

Supplementary Table 2: Top biological functions and canonical pathways of AQP1 according to an Ingenuity Pathway Analysis

**Top biological functions**		
**Diseases and disorders**		
**Name**	**P value**	**Number of molecules**
Cancer	5.74E-04–9.13E-12	3515
Organismal injury and abnormalities	5.74E-04–9.13E-12	3594
Reproductive system disease	5.74E-04–9.13E-12	1763
Inflammatory response	5.55E-04–9.46E-12	797
Dermatological diseases and conditions	9.81E-05–5.55E-09	233
**Molecular and celluar functions**		
**Name**	**P value**	**Number of molecules**
Cellular movement	5.20E-04–1.14E-09	817
Cellular development	5.88E-04–4.14E-09	953
Cellular growth and proliferation	5.88E-04 –4.14E-09	972
Cell morphology	5.29E-04–1.42E-08	932
Cellular assembly and organization	2.55E-04–1.42E-08	551
**Top canonical pathways**		
**Name**	**P value**	**Number of molecules**
Dendritic cell maturation	7.37E-07	37.8% (65/172)
Communication between innate and adaptive immune cells	2.37E-06	46.5% (33/71)
Antigen presentation pathway	3.07E-06	56.8% (21/37)
Graft-versus-host disease signaling	2.41E-05	52.6% (20/38)
Role of pattern recognition receptors in recognition of bacteria and viruses	3.15E-05	37.2% (48/129)

Supplementary Table 3: Backgrounds of 50 ESCC patients

**Sex**		
Male	42	84%
Female	8	16%
**Age**		
average	62.4	
<65	30	60%
≥65	20	40%
**Smoking (BI)**		
Non smoking	10	20%
<1200	14	28%
≥1200	16	32%
N.A	10	20%
**Drinking (ethanol: g/day)**		
Non drinking	14	28%
Opportunity	5	10%
drink	17	34%
<60	13	26%
≥60	1	2%
N.A		
**Histology type**		
Well/Moderate	35	70%
Poor	15	30%
**Location**		
Ce-Ut	5	10%
Mt	24	48%
Lt-Ae	21	42%
**Tumor size (mm)**		
<50	31	62%
≥50	19	38%
**Lymphatic invasion**		
Negative	22	44%
Positive	28	56%
**Venous invasion**		
Negative	26	52%
Positive	24	48%
**pT**		
pT1	19	38%
pT2-4	31	62%
**pN**		
pN0	19	38%
pN1-3	31	62%
**pStage**		
1	9	18%
2	25	50%
3	13	26%
4	3	6%
**Postoperative therapy**		
Chemotherapy	31	62%
Radiation	11	22%
**Reccurrence**		
Positive	20	40%
Negative	30	60%

BI:Brinkmann index
pT: pathological T stage, pN: pathological N stage
[*] $p<0.05$: Fisher's exact test.

Supplementary Table 4: 5-year overall survival rate with each cut-off values for cytoplasm and nuclear membrane respectively

Population	*Cytoplasm*			*Nuclear membrane*		
Cut-off value	5-year overall survival rate		*P* value	5-year overall survival rate		*P* value
	Low group	High group		Low group	High group	
10	81.2% (n=16)	62.8% (n=34)	0.235	62.7% (n=19)	72.7% (n=31)	0.495
20	76.0% (n=25)	61.5% (n=25)	0.305	63.6% (n=28)	75.5% (n=22)	0.457
30	70.3% (n=31)	66.5% (n=19)	0.996	61.0% (n=35)	86.6% (n=15)	0.121
40	70.8% (n=35)	65.0% (n=15)	0.919	63.6% (n=40)	90.0% (n=10)	0.168

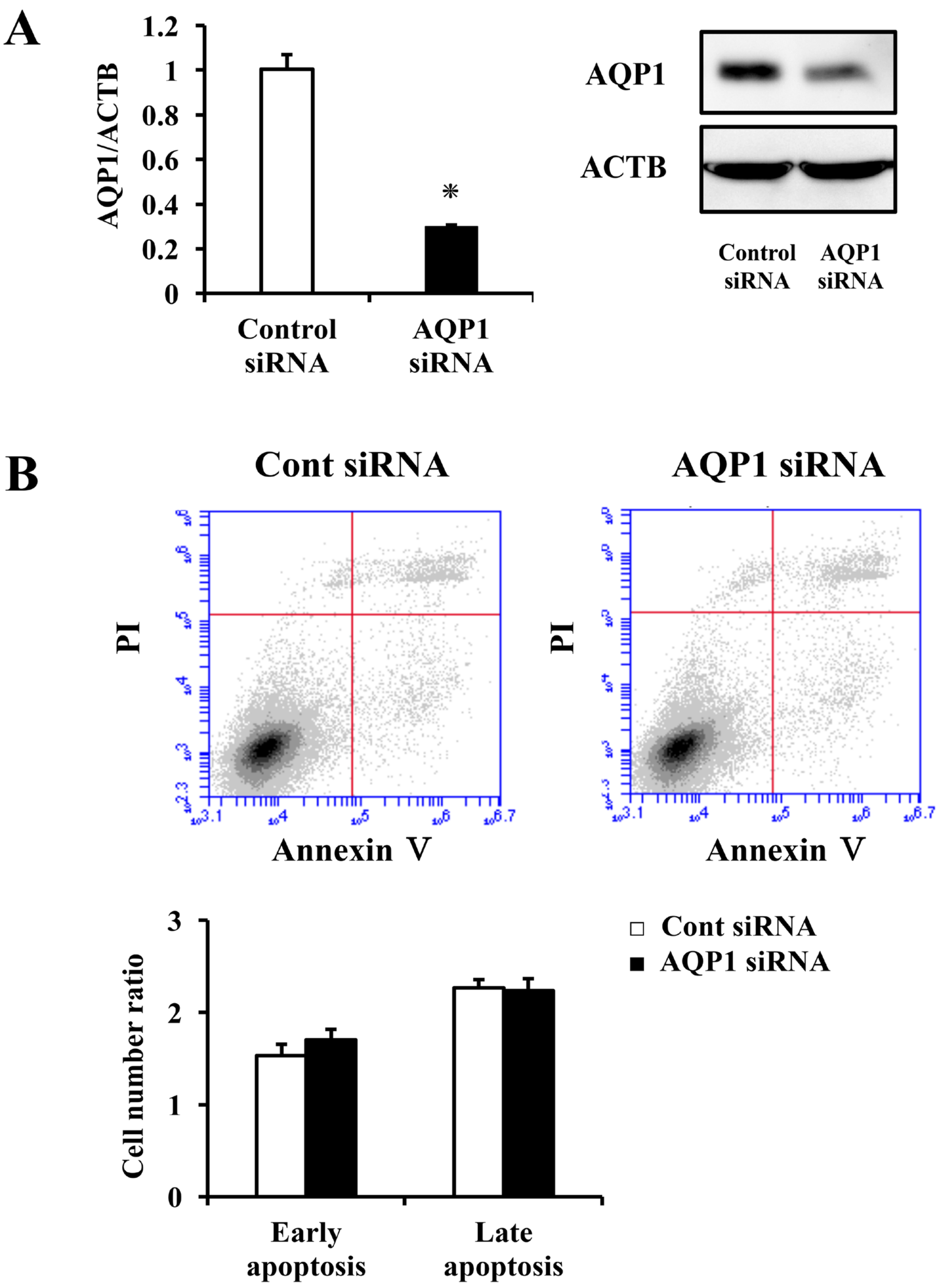

Supplementary Figure 1: AQP1 has no regulation of the cell survival in KYSE70 cells. (A) AQP1 siRNA effectively reduced AQP1 mRNA levels in KYSE70 cells. Mean ± SEM. n = 3. $^{*}p < 0.05$ (significantly different from control siRNA). Western blotting revealed that AQP1 siRNA effectively reduced AQP1 protein levels in KYSE70 cells. **(B)** AQP1 had no influence on apoptosis in KYSE70 cells. Apoptosis was determined by flow cytometry using PI/Annexin V double staining. Mean ± SEM. n = 3. $^{*}p < 0.05$ (significantly different from control siRNA).

A ***Esophageal adenocarcinoma***

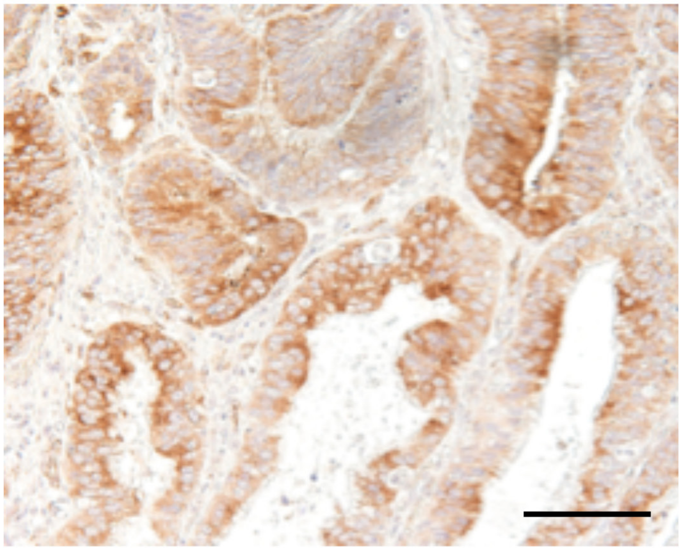

B ***Esophageal basaloid cell carcinoma***

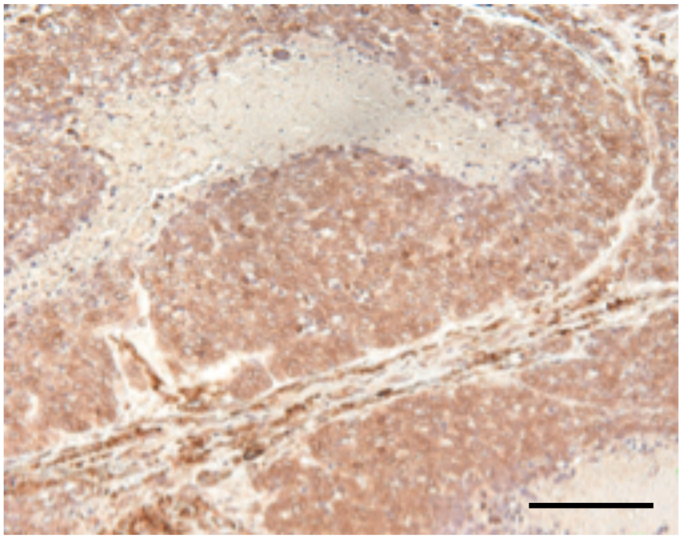

Supplementary Figure 2: AQP1 protein expression in human esophageal adenocarcinoma and esophageal basaloid-type SCC. (A) Immunohistochemical staining of human esophageal adenocarcinoma using an anti-AQP1 antibody. AQP1 was expressed in esophageal adenocarcinoma. Magnification: ×200. Bar 100 µm. **(B)** Immunohistochemical staining of human esophageal basaloid-type SCC using an anti-AQP1 antibody. AQP1 was expressed in esophageal basaloid-type SCC. Magnification: ×200. Bar 100 µm.
